# LncRNA DLEU2 accelerates the tumorigenesis and invasion of non–small cell lung cancer by sponging miR‐30a‐5p

**DOI:** 10.1111/jcmm.14749

**Published:** 2019-11-13

**Authors:** Weiming Wu, Yonghong Zhao, Erji Gao, Yang Li, Xiang Guo, Tiancheng Zhao, Weiwei He, Huibiao Zhang

**Affiliations:** ^1^ Department of Thoracic Surgery Shanghai Jiao Tong University Affiliated Sixth People’s Hospital Shanghai China; ^2^ Department of Thoracic Surgery Huadong Hospital Fudan University Shanghai China

**Keywords:** DLEU2, growth, invasion, miR‐30a‐5p, NSCLC

## Abstract

Long non‐coding RNAs (lncRNAs) have been reported to participate in the pathogenesis of non–small cell lung cancer (NSCLC). However, how lncRNA deleted in lymphocytic leukaemia 2 (DLEU2) contributes to NSCLC remains undocumented. The clinical significance of lncRNA DLEU2 and miR‐30a‐5p expression in NSCLC was analysed by using fluorescence in situ hybridization and TCGA cohorts. Gain‐ and loss‐of‐function experiments as well as a NSCLC tumour model were executed to determine the role of lncRNA DLEU2 in NSCLC. DLEU2‐sponged miR‐30a‐5p was verified by luciferase reporter, and RIP assays. Herein, the expression of lncRNA DLEU2 was elevated in NSCLC tissues, and its high expression or low expression of miR‐30a‐5p acted as an independent prognostic factor of poor survival and tumour recurrence in NSCLC. Silencing of lncRNA DLEU2 repressed the tumorigenesis and invasive potential of NSCLC, whereas re‐expression of lncRNA DLEU2 showed the opposite effects. Furthermore, lncRNA DLEU2 harboured a negative correlation with miR‐30a‐5p expression in NSCLC tissues and acted as a sponge of miR‐30a‐5p, which reversed the tumour‐promoting effects of lncRNA DLEU2 by targeting putative homeodomain transcription factor 2 in NSCLC. Altogether, lncRNA DLEU2 promoted the tumorigenesis and invasion of NSCLC by sponging miR‐30a‐5p.

## 1 INTRODUCTION

Non–small cell lung cancer (NSCLC), a subgroup of cancer types, accounts for 80% of lung cancer cases and is associated with the higher mortality of the patients worldwide. 1, 2, 3 Strenuous attempts have been made to improve the treatment of NSCLC, but the survival outcomes of the patients are still undesirable owing to its aggressiveness and distant metastasis.^4^ Therefore, identification of promising cancer‐related biomarkers may offer strategies to highlight the early detection of NSCLC.

The maladjustment of long non‐coding RNAs (lncRNAs) is involved in the pathogenesis of NSCLC.^5^, ^6^, ^7^ For one thing, lncRNAs can be utilized as the indicators for forecasting the prognosis of NSCLC.^6^, ^8^, ^9^, ^10^ Raised expression of lncRNA AFAP1‐AS1^10^ or down‐regulation of EPB41L4A‐AS2 6 indicates an unfavourable prognosis in patients with NSCLC. For another, lncRNAs function as oncogenes^11^, ^12^ or anti‐oncogenes in NSCLC.^6^, ^13^ LncRNA AFAP1‐AS1, linc00460 and LINC00312 facilitate the proliferation, invasion and epithelial‐mesenchymal transition,^5^, ^11^, ^12^ while EPB41L4A‐AS2^6^ and NKILA^13^ have the opposite effects in NSCLC cells.

In addition, lncRNAs act as the sponges of miRNAs in NSCLC. LncRNA uc.339 accelerates the carcinogenesis by regulating miRNAs.^14^ lncRNA GIHCG and linc00673 function as oncogenic factors by sponging miR‐200b/a/429/‐150‐5p,^15^, ^16^ whereas inhibition of DGCR5 favours the radio‐sensitivity in laryngeal carcinoma by sponging miR‐195.^17^

Herein, we found that increased expression of lncRNA DLEU2 or reduced expression of miR‐30a‐5p was associated with poor prognosis in patients with NSCLC. LncRNA DLEU2 promoted the proliferation and invasion and harboured a negative correlation with miR‐30a‐5p expression in NSCLC tissues. Moreover, lncRNA DLEU2 acted as a sponge of miR‐30a‐5p, which reversed lncRNA DLEU2–induced cell proliferation by targeting putative homeodomain transcription factor 2 (PHTF2) in NSCLC.

## 2 MATERIALS AND METHODS

### 2.1 Materials

NSCLC cell lines (A549 and NCI‐H460) used in our study were purchased from the Institute of Chemistry and Cell Biology. Lentivirus‐mediated sh‐DLEU2 and negative control (sh‐NC) vectors were purchased from Genechem. LncRNA DLEU2 plasmid, pcDNA3.1 and miR‐30a‐5p mimic or inhibitor were from GenePharma. The antibodies against PCNA, MMP‐2 and PHTF2 were from Abcam.

### 2.2 Clinical samples

The RNA‐seq data including the clinicopathological and prognostic information of NSCLC and the expression levels of lncRNA DLEU2, miR‐30a‐5p/‐30b‐5p/‐30c‐5p/‐30d‐5p/‐30e‐5p and PHTF2 were downloaded from the TCGA dataset. A tissue microarray (TMA) containing 20 paired lung adenocarcinoma (LAC) tissues (Cat No. ZL‐LUC1601) was from Superbiotek, and another 15 paired frozen LAC tissue samples were from our hospital. These protocols were approved by the ethics committee of our hospital.

### 2.3 Plasmid construction

The wild‐type (WT) DLEU2 and PHTF2 3′ untranslated region (UTR) vectors, which contained miR‐30a‐5p‐specific binding sites, and the mutant (Mut) lncRNA DLEU2 and PHTF2 3′UTR vectors, containing the Mut miR‐30a‐5p binding sites, were synthesized from GenePharma. Additionally, sh‐DLEU2, a shRNA that targets lncRNA DLEU2 transcription (GCTTACACTTATGGAGCTA), and a negative control si‐NC (GCTCACATTG GTGATA CTA) were constructed by GenePharma.

### 2.4 Bioinformatic analysis

LncRNA DLEU2–specific binding with miRNAs and the targets of miR‐30a‐5p were identified using the StarBase v2.0 on the basis of the high stringency (>5) and the number of cancers types (≥5).

### 2.5 RNA fluorescence in situ hybridization

Oligonucleotide‐modified probe sequences for lncRNA DLEU2 (5′GAAAGTGCTT CTTTCCTCGAGAA3′‐FAM) and miR‐30a‐5p (5′ CTTCCAGTCGAGGATGTTTAC A3′) were synthesized for fluorescence in situ hybridization (FISH) analysis, which was performed as previously described.^18^

### 2.6 Cell culture, transfection and quantitative real‐time PCR

The expression levels of lncRNA DLEU2 in NSCLC cell lines and tissue samples were detected by quantitative real‐time PCR (qRT‐PCR) analysis. Cell culture, transfection and qRT‐PCR analysis were carried out as previously described.^18^ The primers used are listed in Table S1.

### 2.7 Western blot analysis

Western blot analysis was implemented as previously described.^18^ Primary antibodies against PCNA (ab19166, Rabbit polyclonal, Abcam), MMP‐2 (ab92536, Rabbit monoclonal, Abcam) and PHTF2 (ab107966, Rabbit polyclonal, Abcam) were diluted according to the instructions and incubated overnight at 4°C.

### 2.8 Cell viability and transwell invasion assays

Cell viability and Transwell invasion assays were conducted as previously described.^18^

### 2.9 Dual‐luciferase report and RNA immunoprecipitation assays

Luciferase gene report and RNA immunoprecipitation (RIP) assays were executed according to the previous report.^19^

### 2.10 Animal experiments

Six‐week‐old female BALB/c‐nu mice were injected subcutaneously with 5 × 10^6^ A549 cells stably transfected with sh‐DLEU2 or sh‐NC and were monitored daily so as to develop a subcutaneous xenograft tumour. The detailed detection measures were performed as previously described.^18^ This animal study was approved by the ethics committee of our hospital.

### 2.11 Haematoxylin and eosin and Immunohistochemistry analysis

Xenograft tumour tissues were harvested and fixed in 4% paraformaldehyde and preserved in optimal cutting temperature compound. The tumour tissues were stained with haematoxylin and eosin (HE) for the histological analysis, and immune‐stained for Ki‐67 (Abcam) as previously described.^18^

### 2.12 Statistical analysis

Statistical analysis was carried out as previously described.^19^

## 3 RESULTS

### 3.1 Elevated expression of lncRNA DLEU2 is associated with poor survival in patients with NSCLC

**Figure 1:**
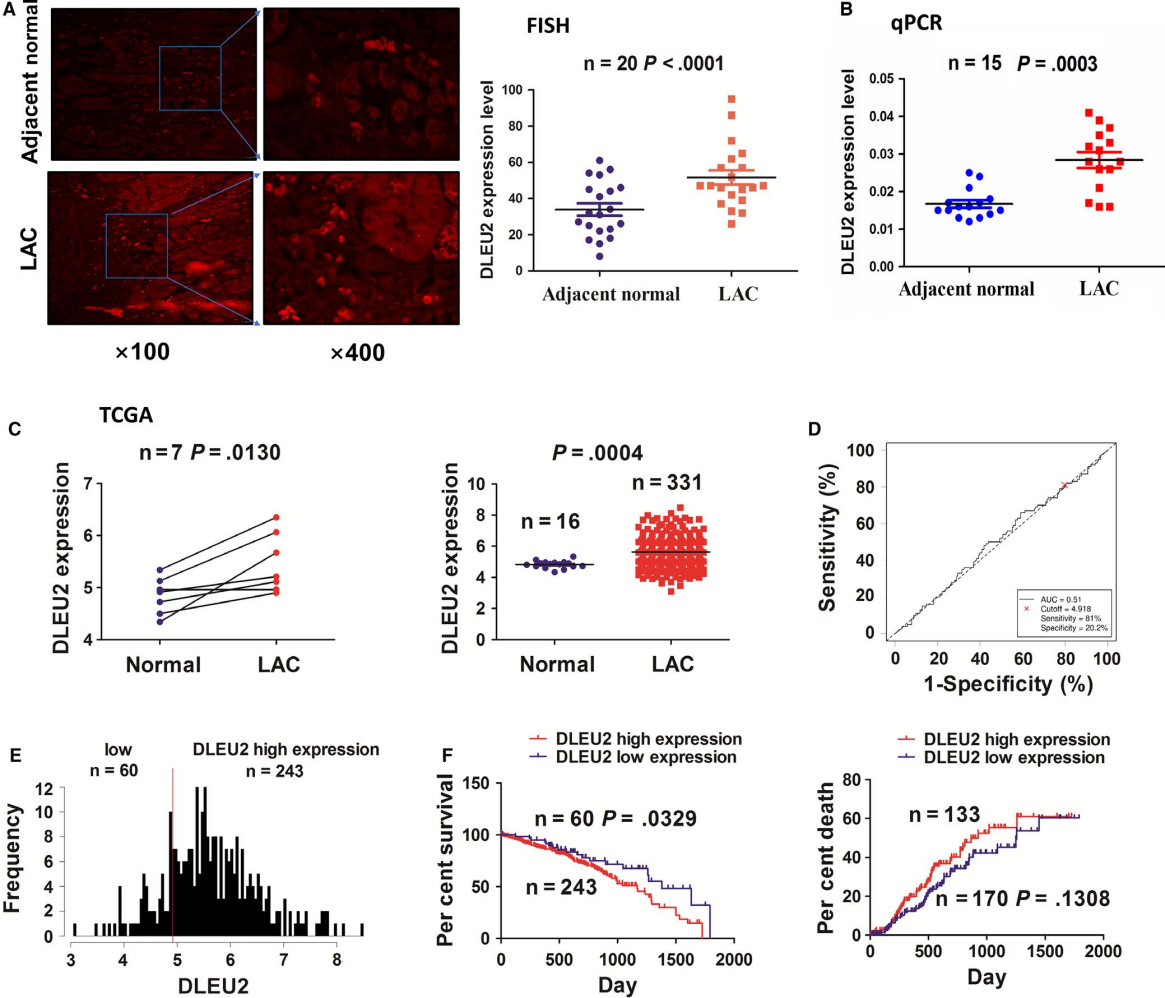
Up‐regulation of lncRNA DLEU2 expression was associated with poor survival in patients with NSCLC. A, FISH analysis of the localization and expression levels of lncRNA DLEU2 in LAC tissues. B, qRT‐PCR analysis of the expression levels of lncRNA DLEU2 in LAC tissue samples. C, TCGA analysis of the expression levels of lncRNA DLEU2 in paired and unpaired LAC tissues. D, ROC curve analysis of the cut‐off value, AUC, sensitivity and specificity of lncRNA DLEU2 in LAC patients. E, The cut‐off value divided the LAC patients into high DLEU2 expression and low DLEU2 expression groups. F, Kaplan‐Meier analysis of the association of high or low lncRNA DLEU2 expression with the poor survival and tumour recurrence in LAC patients

As shown by FISH analysis, we found that lncRNA DLEU2 expression, predominantly in the cytoplasm, was dramatically raised in lung adenocarcinoma (LAC) tissue samples compared with the normal tissues (n = 20, *P* < .0001; Figure 1A). qRT‐PCR analysis also showed the up‐regulation of lncRNA DLEU2 in LAC tissue samples as compared with the adjacent normal tissues (n = 15, *P* = .0003; Figure 1B). These results were further validated in paired (n = 7, *P* = .013) and unpaired LAC tissues (n = 331, *P* = .0004) in TCGA cohort (Figure 1C).

To analyse the association between lncRNA DLEU2 expression and clinicopathological characteristics and prognosis in patients with LAC based on the lncRNA DLEU2 expression levels, survival time and survival status, we obtained the cut‐off value (4.918), AUC (0.51), sensitivity (81.0%) and specificity (20.2%) of DLEU2 in LAC patients (Figure 1D), suggesting that lncRNA DLEU2 might be a potential marker for LAC patients.

According to the cut‐off value of lncRNA DLEU2, the patients were divided into high DLEU2 expression group (n = 243) and low DLEU2 expression group (n = 60) (Figure 1E). High expression of lncRNA DLEU2 had no association with the clinicopathological factors in LAC patients (each *P* > .05; Table S2). Subsequently, as indicated by Kaplan‐Meier analysis, we found that the patients with high DLEU2 expression had a poorer survival (*P* = .0329), but showed no difference in tumour recurrence (*P* = .1308), as compared with those with low DLEU2 expression (Figure 1F). According to the univariate and multivariate analysis, we found that high DLEU2 expression as well as lymph node metastasis was an independent prognostic factor of poor survival in LAC patients (Tables S3).

### 3.2 lncRNA DLEU2 enhances the proliferation and invasion of NSCLC cells

**Figure 2:**
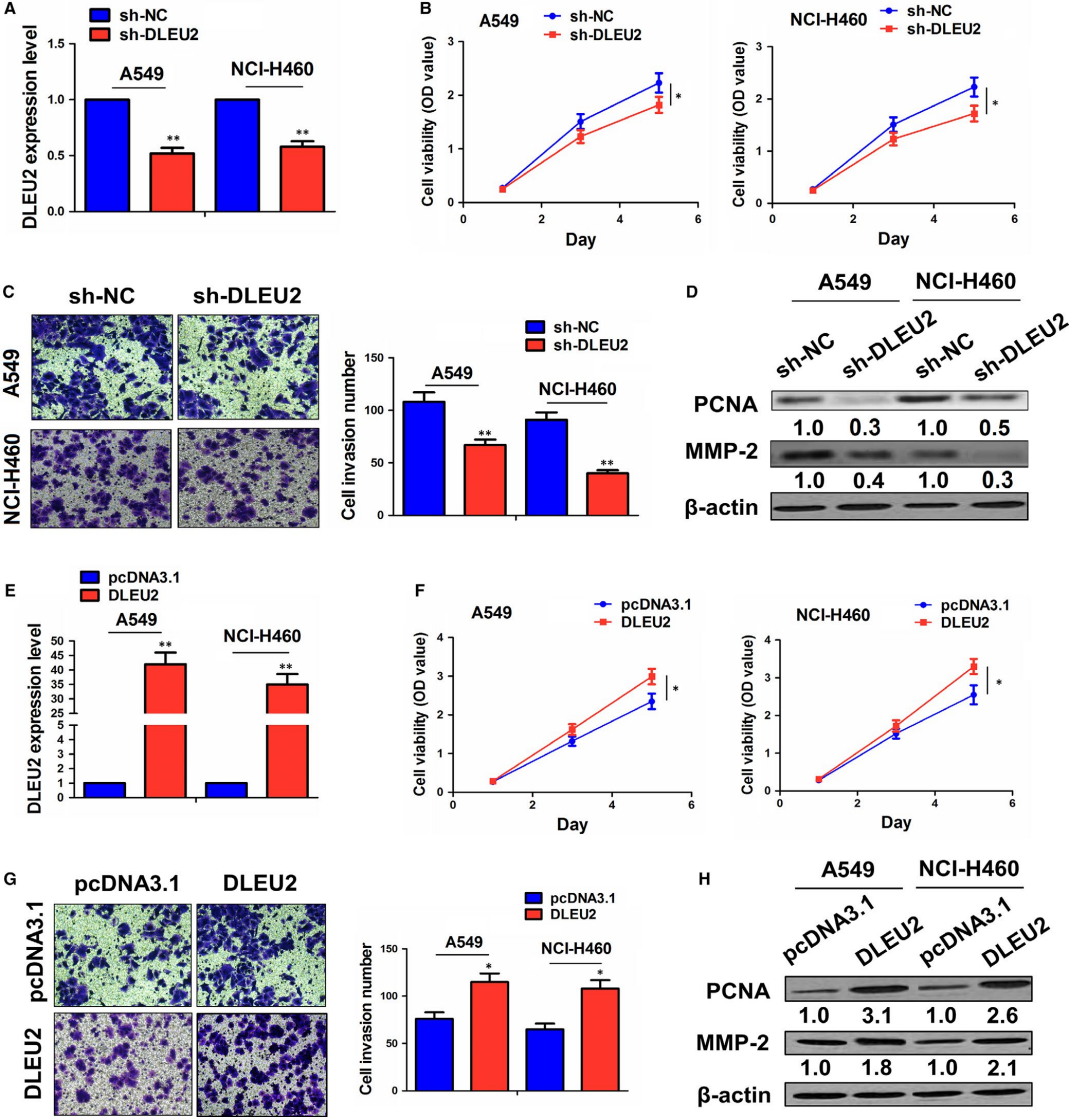
LncRNA DLEU2 promoted the proliferation and invasion of NSCLC cells. A, qRT‐PCR analysis of the knockdown effect of sh‐DLEU2 in A549 and NCI‐H460 cell lines. B, MTT analysis of the proliferation viability after the transfection of sh‐DLEU2 in A549 and NCI‐H460 cells. C, Transwell analysis of the invasive potential after the transfection of sh‐DLEU2 in A549 and NCI‐H460 cells. D, Western blot analysis of the expression levels of PCNA and MMP‐2 after the transfection of sh‐DLEU2 in A549 and NCI‐H460 cells. (E) qRT‐PCR analysis of the overexpression effect of lncRNA DLEU2 plasmid in A549 and NCI‐H460 cells. F, MTT analysis of the proliferation viability after the transfection of lncRNA DLEU2 plasmid in A549 and NCI‐H460 cells. G, Transwell analysis of the invasive potential after the transfection of lncRNA DLEU2 plasmid in A549 and NCI‐H460 cells. H, Western blot analysis of the expression levels of PCNA and MMP‐2 after the transfection of lncRNA DLEU2 plasmid in A549 and NCI‐H460 cells. Data are the mean ± SEM of three experiments. * *P* < .05; ** *P* < .01

The knockdown effect of sh‐DLEU2 vector in A549 and NCI‐H460 cell lines was determined by qRT‐PCR analysis (Figure 2A). We found that knockdown of DLEU2 reduced the proliferative viability (Figure 2B) and invasive potential (Figure 2C) in A549 and NCI‐H460 cells. In addition, the expression levels of PCNA and MMP‐2, as evaluated by Western blot analysis, were decreased by knockdown of lncRNA DLEU2 in these two cell lines (Figure 2D).

Then, the overexpression effect of lncRNA DLEU2 plasmid in these two lines was assessed by qRT‐PCR analysis (Figure 2E). Overexpression of lncRNA DLEU2 promoted the proliferative viability (Figure 2F) and invasive potential (Figure 2G) in A549 and NCI‐H460 cells. The expression levels of PCNA and MMP‐2, as estimated by Western blot analysis, were increased by overexpression of lncRNA DLEU2 in these two cells (Figure 2H).

### 3.3 MiR‐30a‐5p displays a negative correlation with lncRNA DLEU2 expression in NSCLC tissues

**Figure 3:**
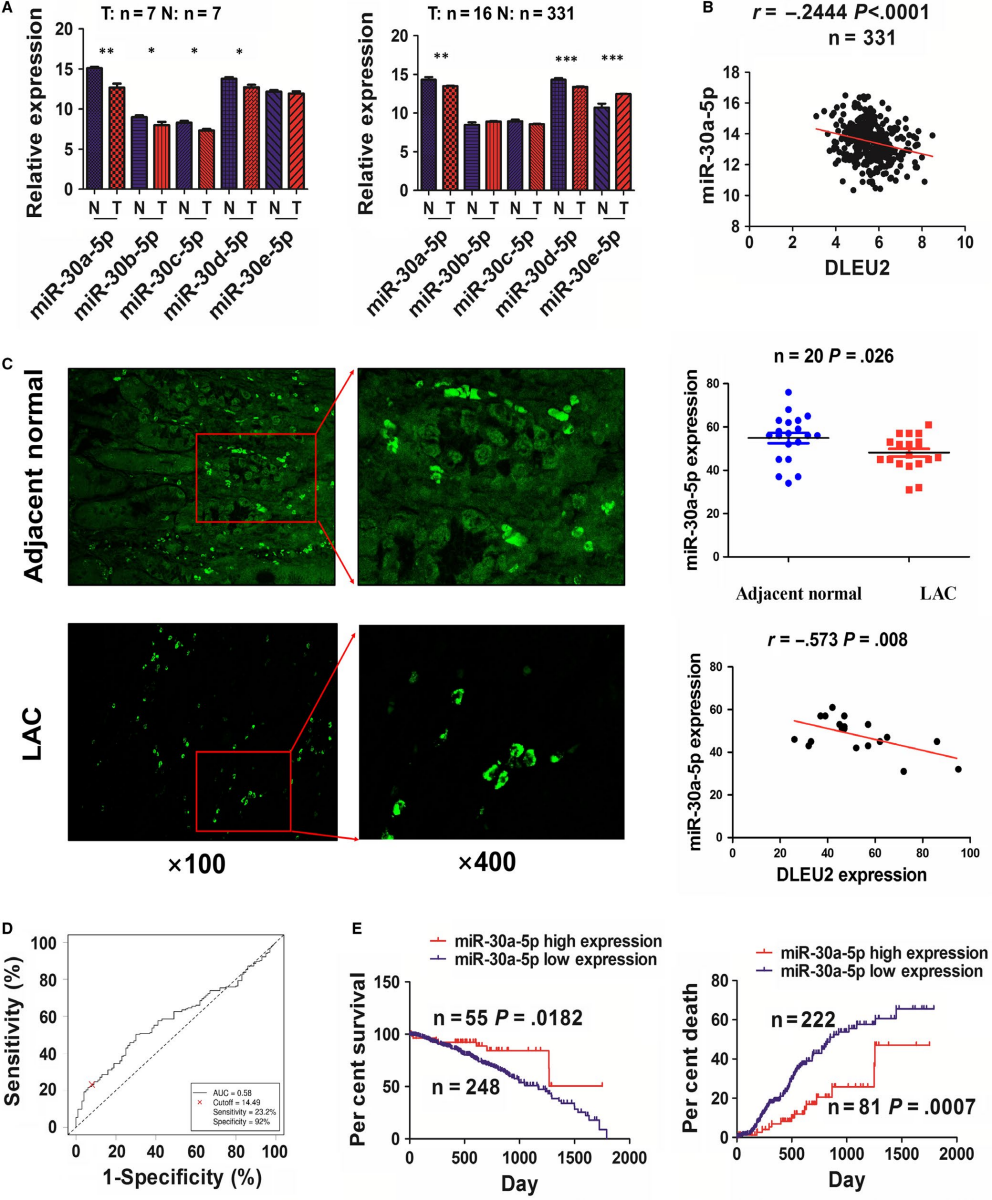
MiR‐30a‐5p possessed a negative correlation with lncRNA DLEU2 expression in NSCLC. A, TCGA analysis of the expression levels of miRNAs in paired and unpaired LAC tissues. B, Pearson analysis of the correlation of lncRNA DLEU2 with miR‐30a‐5p expression in LAC tissues. C, FISH analysis of the localization and expression levels of miR‐30a‐5p and validation of the correlation of lncRNA DLEU2 with miR‐30a‐5p expression in LAC tissues. D, ROC analysis of the cut‐off value, AUC, sensitivity and specificity of miR‐30a‐5p in LAC patients. E, Kaplan‐Meier analysis of the association of high or low miR‐30a‐5p expression with poor survival and tumour recurrence in LAC patients. Data are the mean ± SEM of three experiments. * *P* < .05, ** *P* < .01 and *** *P* < .0001

To elucidate the mechanisms by which lncRNA DLEU2 contributes to NSCLC, we used a starBasev2.0 tool to screen five miRNAs that may have the strongest binding potential with lncRNA DLEU2 in cancers (Table S4). The expression levels of these miRNAs were detected in LAC tissues using the TCGA data set. We found that miR‐30a‐5p/‐30d‐5p rather than other miRNAs had a decreased expression in paired and unpaired LAC tissues (Figure 3A). Pearson correlation analysis showed that miR‐30a‐5p harboured a most significantly negative correlation with lncRNA DLEU2 expression (*r* = −0.2444, *P* < .0001; Figure 3B), as compared with other miRNAs in LAC tissues (Figure S1). As shown by FISH analysis, we also validated that miR‐30a‐5p, mainly localized in the cytoplasm, was down‐regulated (*P* = .026) and had a negative correlation with lncRNA DLEU2 expression in LAC tissues (*r* = −0.573, *P* = .008; Figure 3C).

We further obtained a cut‐off value (14.49), AUC (0.58), sensitivity (23.2%) and specificity (92%) of miR‐30a‐5p in LAC patients (Figure 3D) and divided the patients into high and low miR‐30a‐5p expression groups (Figure S2). As indicated in Table S5, low expression of miR‐30a‐5p had an association with lymph node metastasis (*P* = .005), rather than other parameters in LAC (each *P* > .05). Subsequently, Kaplan‐Meier analysis demonstrated that the patients with low miR‐30a‐5p expression possessed a lower survival (*P* = .0182) and a higher tumour recurrence (*P* = .0007) in comparison with those with high miR‐30a‐5p expression (Figure 3E). Univariate and multivariate analysis unveiled that low miR‐30a‐5p expression as well as lymph node metastasis was an independent prognostic factor of poor survival and tumour recurrence in LAC patients (Tables S6,7).

### 3.4 lncRNA DLEU2 acts as a sponge of miR‐30a‐5p in NSCLC cells

**Figure 4:**
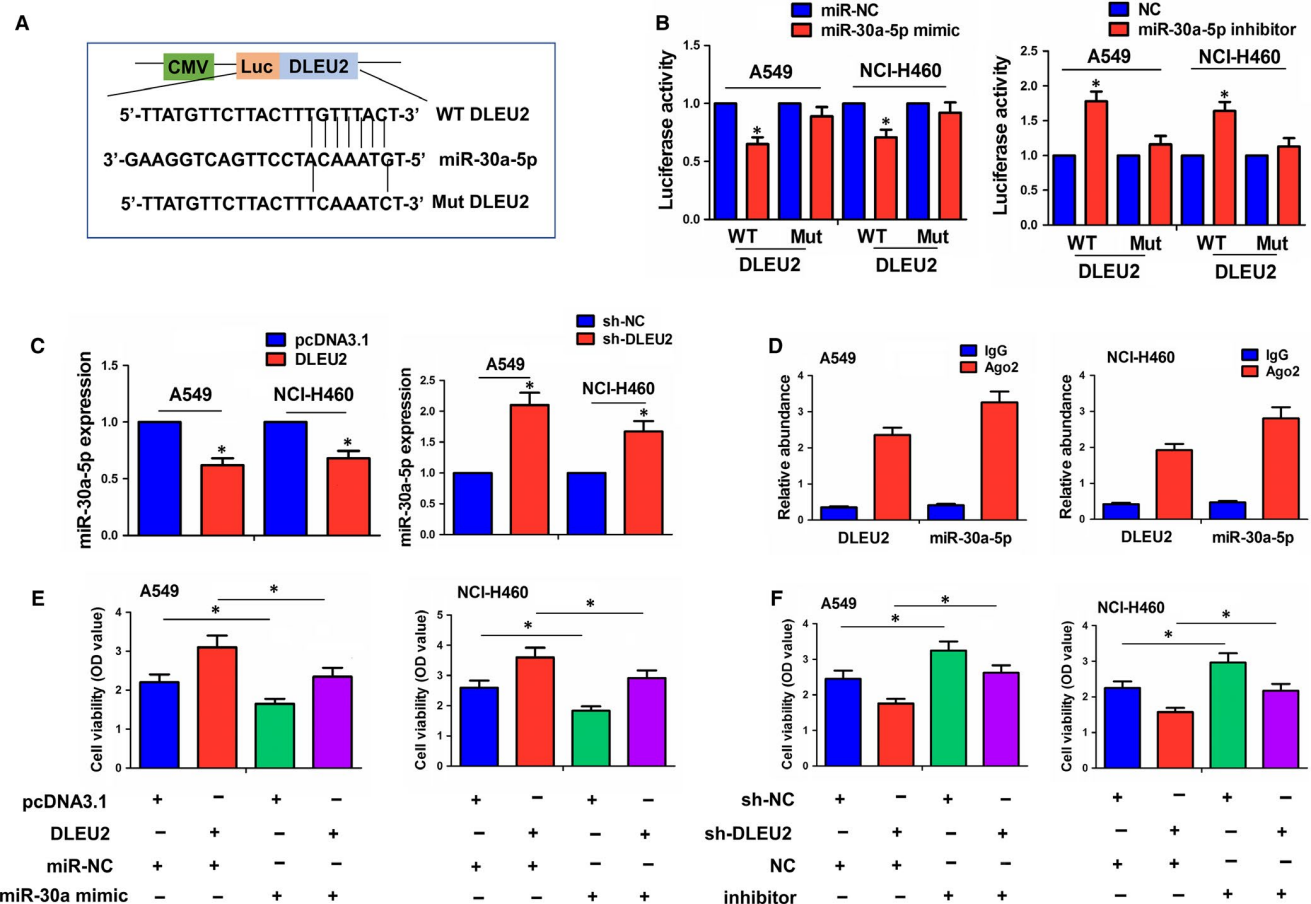
LncRNA DLEU2 acted as a sponge of miR‐30a‐5p in NSCLC cells. A, Schematic representation of the binding sites between miR‐30a‐5p and WT or Mut lncRNA DLEU2. B, Luciferase activity of WT or Mut DLEU2 after the cotransfection with the miR‐30a‐5p mimic or inhibitor and WT or Mut DLEU2 vector in A549 and NCI‐H460 cells. C, qRT‐PCR analysis of the effects of lncRNA DLEU2 on the expression levels of miR‐30a‐5p in A549 and NCI‐H460 cells. D, RIP analysis of the amount of lncRNA DLEU2 and miR‐30a‐5p pulled down from the Ago2 protein in A549 and NCI‐H460 cells (E and F). MTT analysis of the proliferation viability after cotransfection with DLEU2 plasmid and miR‐30a‐5p mimic or sh‐DLEU2 and miR‐30a‐5p inhibitor in A549 and NCI‐H460 cells. Data shown are the mean ± SEM of three experiments. *P < .05

The binding sites between miR‐30a‐5p and WT or Mut DLEU2 are indicated in Figure 4A. We cotransfected A549 and NCI‐H460 cells with WT or Mut DLEU2 reporter and miR‐30a‐5p mimic or inhibitor, and found that miR‐30a‐5p mimic reduced the luciferase activity of WT DLEU2, while miR‐30a‐5p inhibitor reversed this effect (Figure 4B). Re‐expression of lncRNA DLEU2 reduced the expression levels of miR‐30a‐5p, indicated by qRT‐PCR analysis, but knockdown of lncRNA DLEU2 had an opposite effect (Figure 4C). Furthermore, RIP assay was confucted for Ago2 protein in A549 and NCI‐H460 cells, and endogenous lncRNA DLEU2 and miR‐30a‐5p pulled down from Ago2‐expressed cell lines were enriched in Ago2 pellet as compared with the input control (Figure 4D). The miR‐30a‐5p mimic and DLEU2 plasmid or miR‐30a‐5p inhibitor and sh‐DLEU2 vector were cotransfected into A549 and NCI‐H460 cells, indicating that miR‐30a‐5p mimic repressed the proliferative viability and attenuated the proliferation‐promoting effects of lncRNA DLEU2 (Figure 4E), but miR‐30a‐5p inhibitor reversed these effects (Figure 4F).

### 3.5 MiR‐30a‐5p reverses lncRNA DLEU2–induced PHTF2 expression in NSCLC cells

**Figure 5:**
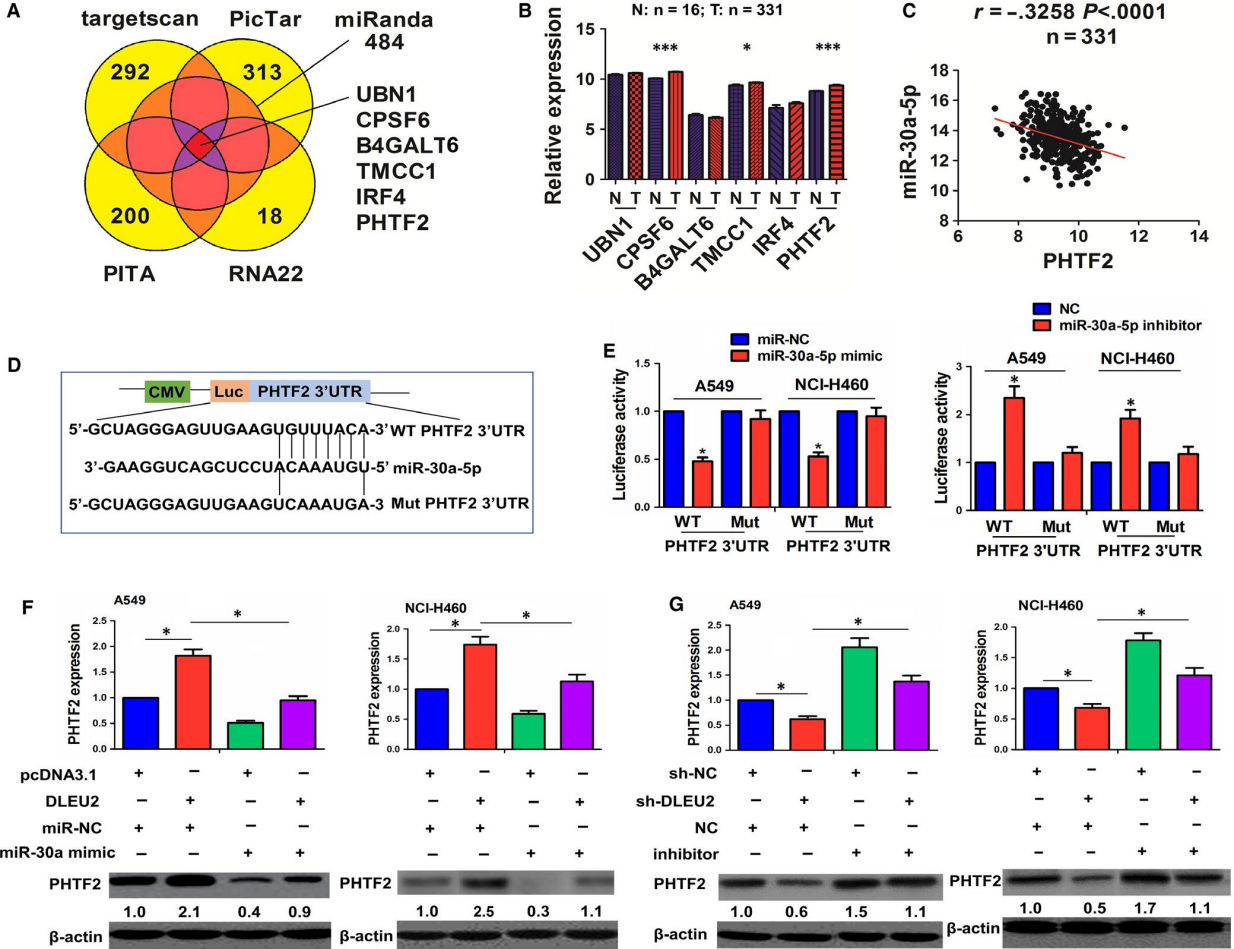
miR‐30a‐5p reversed lncRNA DLEU2–induced PHTF2 expression in NSCLC cells. A, Identification of the targets of miR‐30a‐5p using a starBasev2.0 prediction tool. B, TCGA analysis of the expression levels of these target genes in unpaired LAC tissues. C, Pearson analysis of the correlation of miR‐30a‐5p with lncRNA PHTF2 expression in LAC tissues. D, Schematic representation of the binding sites between miR‐30a‐5p and WT or Mut PHTF2 3’UTR. E, Luciferase activity of WT or Mut PHTF2 3’UTR after the cotransfection with miR‐30a‐5p mimic or inhibitor and WT or Mut PHTF2 3’UTR in A549 and NCI‐H460 cells (F and G). qRT‐PCR and western blot analysis of the expression levels of PHTF2 after cotransfection with lncRNA DLEU2 plasmid and miR‐30a‐5p mimic or sh‐DLEU2 and miR‐30a‐5p inhibitor in A549 and NCI‐H460 cells. Data shown are the mean ± SEM of three experiments. *P < .05 and ***P < .0001

A starBasev2.0 tool was used to screen six targets of miR‐30a‐5p (UBN1, CPSF6, B4GALT6, TMCC1, IRF4 and PHTF2) (Figure 5A). We found that CPSF6, TMCC1 and PHTF2 had an increased expression in paired (Figure S3A) and unpaired LAC tissues (Figure 5B). Pearson correlation analysis showed that PHTF2 rather than CPSF6 and TMCC1 had a most obviously negative correlation with miR‐30a‐5p expression in LAC tissues (Figure 5C and Figure S3B). We further obtained a cut‐off value (10.31), AUC (0.52), sensitivity (10.5%) and specificity (94.22%) of PHTF2 in LAC patients and divided the patients into high and low expression groups (Figure S4A). As shown in Table S8, high expression of PHTF2 had no association with the clinicopathological factors in LAC patients (each *P* > .05). Kaplan‐Meier analysis demonstrated that the patients with high PHTF2 expression had a lower survival (*P* = .042) but showed no difference in tumour recurrence (*P* = .615; Figure S4B). Univariate and multivariate analysis uncovered that high PHTF2 expression was not an independent prognostic factor of poor survival in LAC patients (Tables S9).

The binding sites between miR‐30a‐5p and the WT or Mut PHTF2 3’UTR are demonstrated in Figure 5D. We cotransfected A549 and NCI‐H460 cell lines with WT or Mut PHTF2 3’UTR and miR‐30a‐5p mimic or inhibitor, and found that miR‐30a‐5p mimic decreased the luciferase activity of WT PHTF2 3′UTR, while miR‐30a‐5p inhibitor showed the opposite effects. Both of miR‐30a‐5p mimic and inhibitor had no impact on the luciferase activity of Mut PHTF2 3’UTR as compared with the control group (Figure 5E). qRT‐PCR and Western blot analysis showed that overexpression of DLEU2 increased the expression levels of PHTF2, and miR‐30a‐5p reversed DLEU2‐induced PHTF2 expression (Figure 5F). Knockdown of DLEU2 inhibited the expression levels of PHTF2, and miR‐30a‐5p inhibitor reversed this effect (Figure 5G).

### 3.6 Silencing of lncRNA DLEU2 impedes xenograft tumour growth

**Figure 6:**
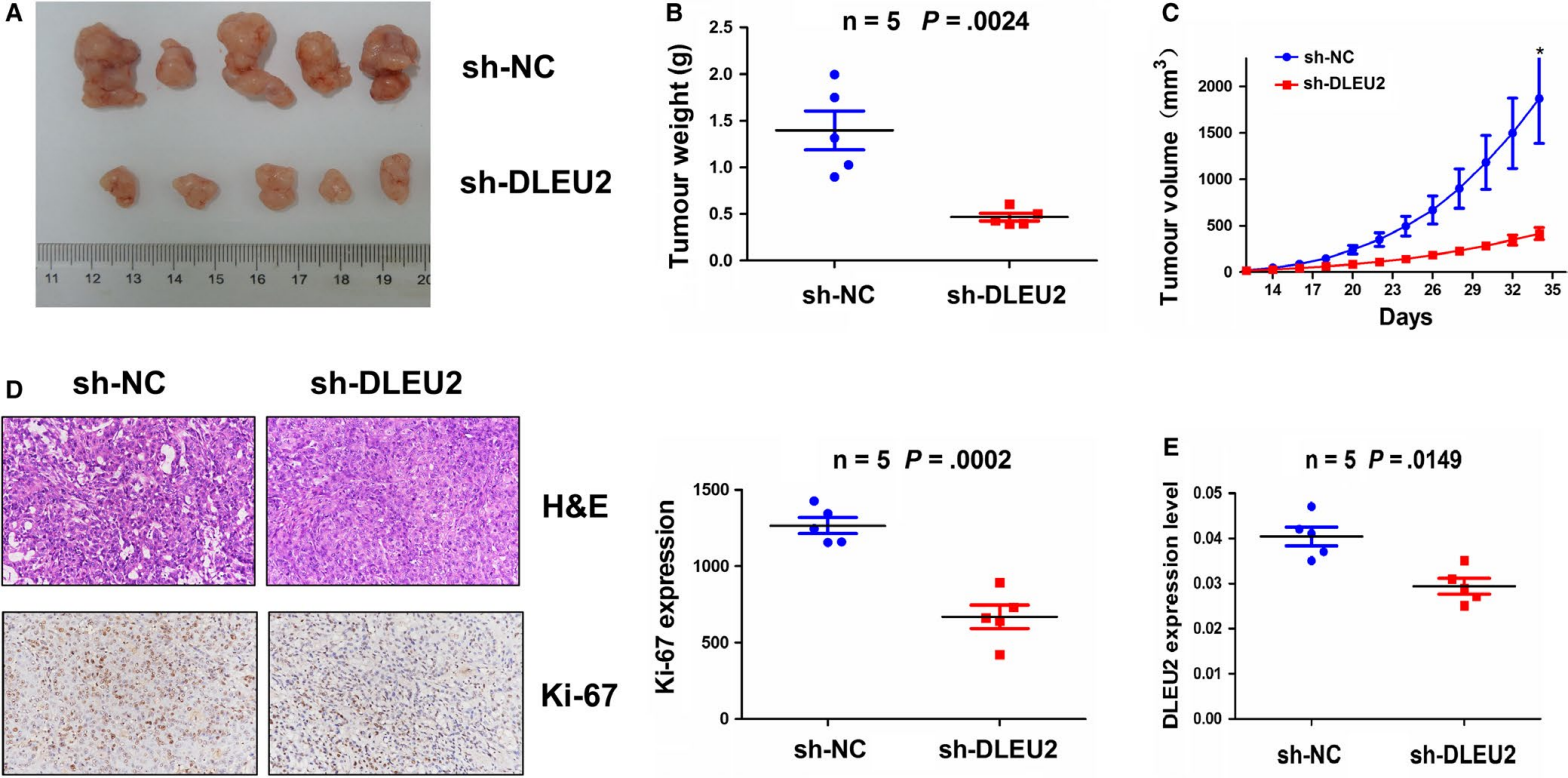
Knockdown of lncRNA DLEU2 inhibited the xenograft tumour growth. A, Representative photographs of the xenograft tumours after the inoculation with sh‐DLEU2 and sh‐NC‐transfected A549 cells. B, Comparison of the average tumour weight between sh‐DLEU2 and sh‐NC groups. C, Growth curve analysis of the tumour proliferation activity after treatment with sh‐DLEU2 and sh‐NC groups. D, HE analysis of the morphological changes for the tumour cell growth between sh‐DLEU2 and sh‐NC groups, and IHC analysis of the expression levels of Ki‐67 in sh‐DLEU2 group. E, qRT‐PCR analysis of the expression levels of lncRNA DLEU2 in sh‐DLEU2 and sh‐NC groups. Data shown are the mean ± SEM of three experiments

A xenograft tumour model was established to observe the tumour growth after subcutaneous inoculation with sh‐DLEU2 stably transfected A549 cells. We found that the proliferative activity of the tumours was lowered in sh‐DLEU2 group as compared with the sh‐NC group (Figure 6A). After the tumour tissues were harvested, the average tumour weight and tumour volumes were reduced in sh‐DLEU2 group (Figure 6B, 6). HE staining showed the morphological changes for the tumour cell growth between sh‐NC and sh‐DLEU2 groups, and mmunohistochemistry (IHC) analysis indicated the decreased expression levels of Ki‐67 in sh‐DLEU2 group (Figure 6D). qRT‐PCR analysis also showed that the expression levels of lncRNA DLEU2 were reduced in sh‐DLEU2 group as compared with the sh‐NC group (Figure 6E).

## 4 DISCUSSION

Accumulating data show that down‐regulation of lncRNA NKILA or up‐regulation of XLOC_008466 and FAL1 is linked to lymph node metastasis and TNM stage,^12^, ^13^, ^15^ and lncRNA AFAP1‐AS1, linc00673 and PVT1 predict a poor prognosis in NSCLC.^10^, ^16^, ^17^ lncRNA DLEU2 is identified to have a differential expression in LAC,^20^ acute myeloid leukaemia^21^ and laryngeal carcinoma.^22^ Herein, we found that lncRNA DLEU2 expression levels were remarkably elevated in NSCLC tissue samples, but had no association with the clinicopathological parameters. High expression of lncRNA DLEU2 was an independent prognostic factor of poor survival in patients with NSCLC.

Functionally, lncRNA DLEU2 inhibits the proliferation, migration and invasion of laryngeal carcinoma^22^ and limits lymphocytic leukaemia.^23^ However, we found that lncRNA DLEU2 promoted the proliferation and invasion of NSCLC cells, but inhibition of lncRNA DLEU2 reversed these effects. PCNA and MMP‐2, tumour proliferation and invasion related markers, act as the poor prognostic factors in NSCLC.^24^ We found that lncRNA DLEU2 induced their expression levels and acted as an oncogene in NSCLC.

Mechanistically, lncRNA XLOC_008466 and linc00673 act by sponging miR‐874/‐150‐5p,^15^, ^16^ but lncRNA PVT1 reduces the proliferation of NSCLC by sponging miR‐195.^17^ Moreover, loss of lncRNA DLEU2 favours the proliferation by regulating miR‐15a/‐16‐1 in chronic lymphocytic leukaemia.^25^ Herein, lncRNA DLEU2 had the potential to bind with the Ago2‐miR‐30a‐5p complex and reduced the expression levels of miR‐30a‐5p, indicating that lncRNA DLEU2 might act as a sponge of miR‐30a‐5p in NSCLC cells.

**Figure 7:**
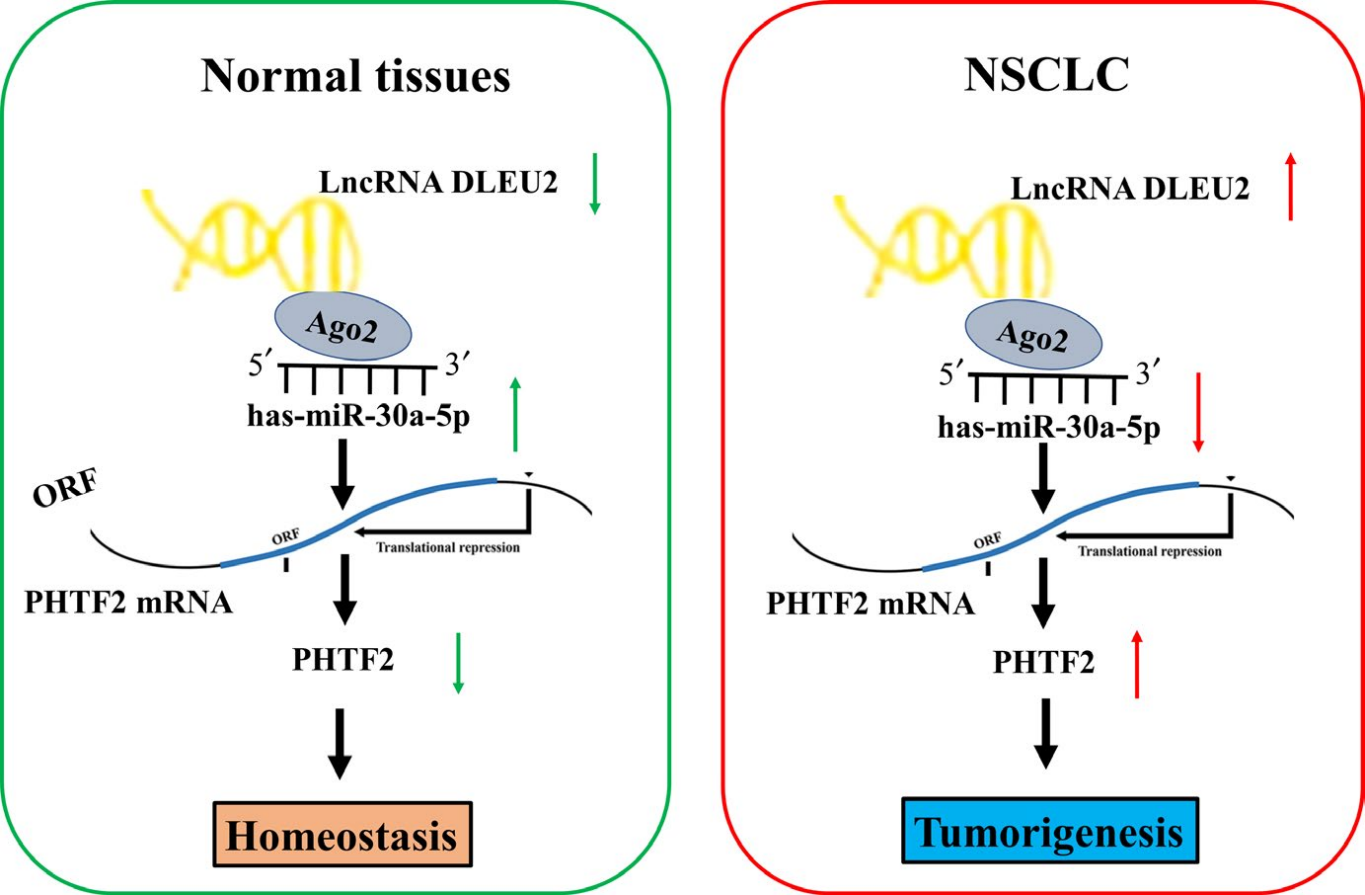
Schematic representation of the underlying mechanism of lncRNA DLEU2/miR‐30a‐5p/PHTF2 axis in NSCLC. DLEU2 sponged miR‐30a‐5p, and thereby up‐regulated PHTF2 expression, contributing to the tumorigenesis of NSCLC

Mounting evidence shows that miR‐30a‐5p represses tumour progression by targeting E2F7^26^ or IGF1R.^27^ Reduced expression of miR‐30a‐5p is a poor indicator in breast cancer.^28^ In accordance, we found that miR‐30a‐5p was down‐regulated in NSCLC tissues, and decreased expression of miR‐30a‐5p was associated with poor survival and tumour recurrence in patients with NSCLC. PHTF2 was further identified as a direct target of miR‐30a‐5p in NSCLC cells and harboured a negative correlation with miR‐30a‐5p expression in NSCLC tissues. MiR‐145‐5p also had a negative correlation with lncRNA DLEU2 expression and attenuated lncRNA DLEU2–induced cell proliferation and PHTF2 expression in NSCLC cells. Our results suggested that lncRNA DLEU2 acted as a sponge of miR‐30a‐5p to promote PHTF2 expression, leading to the tumorigenesis of NSCLC (Figure 7).

Taken together, lncRNA DLEU2 facilitates the tumorigenesis and invasion of NSCLC by sponging miR‐30a‐5p and provides a potential biomarker for predicting the survival of NSCLC.

## CONFLICT OF INTEREST

The authors declare that they have no competing interests.

## AUTHOR CONTRIBUTIONS

Weiwei He and Huibiao Zhang designed this study. Weiming Wu and Yonghong Zhao contributed equally to this article. Weiming Wu, Yonghong Zhao, Erji Gao and Yang Li conducted the experiments. Xiang Guo and Tiancheng Zhao carried out the statistical analysis. Weiming Wu wrote the paper, and Weiwei He revised the paper. All authors read and approved the final manuscript.

## Data Availability

All data used to support the findings of this study are available from the corresponding authors upon request.
