# Supplementary material for: LncRNA DLEU2 accelerates the tumorigenesis and invasion of non–small cell lung cancer by sponging miR‐30a‐5p
Source: J Cell Mol Med. 2019 Nov 13;24(1):441–50. doi: 10.1111/jcmm.14749 (PMC6933340; doi:10.1111/jcmm.14749)
Supplement: Supplementary file 1 [file JCMM-24-441-s001.docx]

**Supplementary Figure legends**

**Fig. S1.** Pearson correlation analysis of the correlation of DLEU2 expression with miRNAs in LAC tissues.

**Fig. S2.** The cutoff value of miR-30a-5p divided the patients into high miR-30a-5p expression group and low miR-30a-5p expression group.

**Fig. S3.** Pearson correlation analysis of the correlation of miR-30a-5p expression with its target genes in LAC tissues. (A) TCGA analysis of the expression levels of the target genes of miR-30a-5p in paired LAC tissues. (B) Pearson correlation analysis of the correlation of miR-30a-5p expression with its target genes in LAC tissues.

**Fig. S4.** Kaplan–Meier analysis of the association of high or low PHTF2 expression with the prognosis of LAC patients. (A) ROC curve analysis of the cutoff value, AUC, sensitivity and specificity of PHTF2 in LAC patients, and the cutoff value of PHTF2 divided the patients into high PHTF2 expression group and low PHTF2 expression group. (B) Kaplan–Meier analysis of the association of high or low PHTF2 expression with poor survival and tumor recurrence in LAC patients.

**Table S1.** Primers used for qRT-PCR reaction

| Markers | Sense (5’-3’) | Antisense (5’-3’) |
| --- | --- | --- |
| DLEU2 | GCGGGTACTTATCTCCGACC | GTTTCCCAGTCGCTGTTCGT |
| β-actin | GCGTGACATTAAGGAGAAGC | CCACGTCACACTTCATGATGG |
| miR-30a-5p | CACTCTCATGTAAACATCCTCGAC | TATGGTTGTTCTGCTCTCTGTGTC |
| U6 | CAGCACATATACTAAAATTGGAACG | ACGAATTTGCGTGTCATCC |
| PHTF2 | AATGGTCCTAGCAAAGATACCCA | CCTGTCCACATGACGACGTAA |

**Table S2** The association of DLEU2 expression with clinicopathologic

characteristics of LAC patients

| Variables | Cases  (n) | DLEU2 | | *P* value |
| --- | --- | --- | --- | --- |
|  |  | High | Low |  |
| Total | 303 | 243 | 60 |  |
| *Age (years)* |  |  |  |  |
| ≥60 | 218 | 175 | 43 |  |
| <60 | 85 | 68 | 17 | 1.000 |
| *Gender* |  |  |  |  |
| Male | 120 | 94 | 26 |  |
| Female | 183 | 149 | 34 | 0.556 |
| *Pathological stage* |  |  |  |  |
| Ⅰ/Ⅱ | 244 | 194 | 50 |  |
| Ⅲ/Ⅳ | 59 | 49 | 10 | 0.591 |
| *T stage* |  |  |  |  |
| T1/T2 | 263 | 213 | 50 |  |
| T3/T4 | 40 | 30 | 10 | 0.396 |
| *N stage* |  |  |  |  |
| Negative | 205 | 165 | 40 |  |
| Positive | 98 | 78 | 20 | 0.878 |
| *M stage* |  |  |  |  |
| Negative | 176 | 137 | 39 |  |
| Positive | 127 | 106 | 21 | 0.245 |

**Table S3** Cox regression analysis of DLEU2 expression as a survival predictor in LAC patients

| Variables | Univariate Cox regression analysis | |  | Multivariate Cox regression analysis | |
| --- | --- | --- | --- | --- | --- |
|  | RR (95% CI) | *P* value |  | RR (95% CI) | *P* value |
| *Age (years)* |  |  |  |  |  |
| ≥ 60 vs. < 60 | 0.980 (0.634 to 1.513) | 0.926 |  | NA | NA |
| *Gender* |  |  |  |  |  |
| Male vs. Female | 1.364 (0.908 to 2.048) | 0.135 |  | NA | NA |
| *Pathological stage* |  |  |  |  |  |
| Ⅲ/Ⅳ vs.Ⅰ/Ⅱ | 2.056 (1.355 to 3.120) | 0.001 |  | 1.467 (0.877 to 2.453) | 0.144 |
| *T stage* |  |  |  |  |  |
| T3+T4 vs. T1+T2 | 1.550 (0.902 to 2.663) | 0.113 |  | NA | NA |
| *N staging* |  |  |  |  |  |
| Positive vs. Negative | 2.155 (1.452 to 3.198) | <0.0001 |  | 1.793 (1.104 to 2.913) | 0.018 |
| *M stage* |  |  |  |  |  |
| Positive vs. Negative | 1.026 (0.686 to 1.535) | 0.901 |  | NA | NA |
| *DLEU2 expression* |  |  |  |  |  |
| High vs. Low | 1.749 (1.040 to 2.940) | 0.035 |  | 1.871 (1.102 to 3.175) | 0.020 |

NA: not analyzed

**Table S4** Identification of DLEU2 specific binding miRNAs

| **name** | **mirAccession** | **lncRNA** | **Target**  **Sites** | **bioComplex** | **clipRead**  **Num** | **Cancer**  **Num** |
| --- | --- | --- | --- | --- | --- | --- |
| [hsa-miR-30e-5p](http://starbase.sysu.edu.cn/starbase2/viewMatureMirInfo.php?table=mirLncRNAInteractionsAll&database=hg19&name=hsa-miR-30e-5p) | MIMAT0000692 | [DLEU2](http://starbase.sysu.edu.cn/starbase2/viewGeneInfo.php?table=mirLncRNAInteractionsAll&database=hg19&name=DLEU2) | 1 | 7 | 118 | 5 |
| [hsa-miR-30c-5p](http://starbase.sysu.edu.cn/starbase2/viewMatureMirInfo.php?table=mirLncRNAInteractionsAll&database=hg19&name=hsa-miR-30c-5p) | MIMAT0000244 | [DLEU2](http://starbase.sysu.edu.cn/starbase2/viewGeneInfo.php?table=mirLncRNAInteractionsAll&database=hg19&name=DLEU2) | 1 | 7 | 118 | 5 |
| [hsa-miR-30a-5p](http://starbase.sysu.edu.cn/starbase2/viewMatureMirInfo.php?table=mirLncRNAInteractionsAll&database=hg19&name=hsa-miR-30a-5p) | MIMAT0000087 | [DLEU2](http://starbase.sysu.edu.cn/starbase2/viewGeneInfo.php?table=mirLncRNAInteractionsAll&database=hg19&name=DLEU2) | 1 | 7 | 118 | 0 |
| [hsa-miR-30b-5p](http://starbase.sysu.edu.cn/starbase2/viewMatureMirInfo.php?table=mirLncRNAInteractionsAll&database=hg19&name=hsa-miR-30b-5p) | MIMAT0000420 | [DLEU2](http://starbase.sysu.edu.cn/starbase2/viewGeneInfo.php?table=mirLncRNAInteractionsAll&database=hg19&name=DLEU2) | 1 | 7 | 118 | 5 |
| [hsa-miR-30d-5p](http://starbase.sysu.edu.cn/starbase2/viewMatureMirInfo.php?table=mirLncRNAInteractionsAll&database=hg19&name=hsa-miR-30d-5p) | MIMAT0000245 | [DLEU2](http://starbase.sysu.edu.cn/starbase2/viewGeneInfo.php?table=mirLncRNAInteractionsAll&database=hg19&name=DLEU2) | 1 | 7 | 118 | 7 |

**Table S5** The correlation of miR-30a-5p expression with clinicopathologic

characteristics of LAC patients

| Variables | Cases  (n) | miR-30a-5p | | *P* value |
| --- | --- | --- | --- | --- |
|  |  | High | Low |  |
| Total | 331 | 57 | 274 |  |
| *Age (years)* |  |  |  |  |
| ≥60 | 237 | 45 | 192 |  |
| <60 | 94 | 12 | 82 | 0.199 |
| *Gender* |  |  |  |  |
| Male | 135 | 23 | 112 |  |
| Female | 196 | 34 | 162 | 1.000 |
| *Pathological stage* |  |  |  |  |
| Ⅰ/Ⅱ | 268 | 51 | 217 |  |
| Ⅲ/Ⅳ | 63 | 6 | 57 | 0.094 |
| *T stage* |  |  |  |  |
| T1/T2 | 290 | 48 | 242 |  |
| T3/T4 | 41 | 9 | 32 | 0.381 |
| *N stage* |  |  |  |  |
| Negative | 226 | 48 | 178 |  |
| Positive | 105 | 9 | 96 | 0.005 |
| *M stage* |  |  |  |  |
| Negative | 194 | 30 | 164 |  |
| Positive | 137 | 27 | 110 | 0.375 |

**Table S6** Cox regression analysis of miR-30a-5p expression as survival predictor

| Variables | Univariate Cox regression analysis | |  | Multivariate Cox regression analysis | |
| --- | --- | --- | --- | --- | --- |
|  | RR (95% CI) | *P* value |  | RR (95% CI) | *P* value |
| *Age (years)* |  |  |  |  |  |
| ≥60 vs. <60 | 1.128 (0.734 to 1.732) | 0.582 |  | NA | NA |
| *Gender* |  |  |  |  |  |
| Male vs. Female | 1.034 (0.701 to 1.526) | 0.866 |  | NA | NA |
| *Pathological stage* |  |  |  |  |  |
| Ⅲ/Ⅳ vs.Ⅰ/Ⅱ | 2.469 (1.650 to 3.695) | <0.0001 |  | 1.676 (1.029 to 2.731) | 0.038 |
| *T stage* |  |  |  |  |  |
| T3+T4 vs. T1+T2 | 1.839 (1.087 to 3.110) | 0.023 |  | NA | NA |
| *N staging* |  |  |  |  |  |
| Positive vs. Negative | 2.404 (1.639 to 3.528) | <0.0001 |  | 1.736 (1.090 to 2.764) | 0.020 |
| *M stage* |  |  |  |  |  |
| Positive vs. Negative | 1.029 (0.695 to 1.522) | 0.887 |  | NA | NA |
| *miR-30a-5p expression* |  |  |  |  |  |
| High vs. Low | 0.320 (0.149 to 0.691) | 0.004 |  | 0.362 (0.168 to 0.874) | 0.010 |

NA: not analyzed

**Table S7** Cox regression analysis of miR-30a-5p expression as recurrence predictor

| Variables | Univariate Cox regression analysis | |  | Multivariate Cox regression analysis | |
| --- | --- | --- | --- | --- | --- |
|  | RR (95% CI) | *P* value |  | RR (95% CI) | *P* value |
| *Age (years)* |  |  |  |  |  |
| ≥60 vs. <60 | 1.007 (0.989 to 1.026) | 0.452 |  | NA | NA |
| *Gender* |  |  |  |  |  |
| Male vs. Female | 1.339 (0.863 to 2.077) | 0.193 |  | NA | NA |
| *Pathological stage* |  |  |  |  |  |
| Ⅲ/Ⅳ vs.Ⅰ/Ⅱ | 0.807 (0.545 to 1.196) | 0.286 |  | NA | NA |
| *T stage* |  |  |  |  |  |
| T3+T4 vs. T1+T2 | 1.455 (0.928 to 2.280) | 0.102 |  | NA | NA |
| *N staging* |  |  |  |  |  |
| Positive vs. Negative | 1.871 (1.111 to 3.150) | 0.019 |  | 1.771 (1.050 to 2.986) | 0.032 |
| *M stage* |  |  |  |  |  |
| Positive vs. Negative | 1.487 (1.012 to 2.185) | 0.043 |  | 1.329 (0.901 to 1.960) | 0.152 |
| *miR-30a-5p expression* |  |  |  |  |  |
| High vs. Low | 0.426 (0.254 to 0.715) | 0.001 |  | 0.452 (0.268 to 0.761) | 0.003 |

NA: not analyzed

**Table S8** The correlation of PHTF2 expression with clinicopathologic

characteristics of LAC patients

| Variables | Cases  (n) | PHTF2 | | *P* value |
| --- | --- | --- | --- | --- |
|  |  | High | Low |  |
| Total | 331 | 24 | 307 |  |
| *Age (years)* |  |  |  |  |
| ≥ 60 | 237 | 12 | 225 |  |
| < 60 | 94 | 12 | 82 | 0.019 |
| *Gender* |  |  |  |  |
| Male | 135 | 11 | 124 |  |
| Female | 196 | 13 | 183 | 0.668 |
| *Pathological stage* |  |  |  |  |
| Ⅰ/Ⅱ | 268 | 18 | 250 |  |
| Ⅲ/Ⅳ | 63 | 6 | 57 | 0.424 |
| *T stage* |  |  |  |  |
| T1/T2 | 290 | 21 | 269 |  |
| T3/T4 | 41 | 3 | 38 | 1.000 |
| *N stage* |  |  |  |  |
| Negative | 226 | 14 | 212 |  |
| Positive | 105 | 10 | 95 | 0.362 |
| *M stage* |  |  |  |  |
| Negative | 194 | 15 | 179 |  |
| Positive | 137 | 9 | 128 | 0.830 |

**Table S9** Cox regression analysis of PHTF2 expression as survival predictor

| Variables | Univariate Cox regression analysis | |  | Multivariate Cox regression analysis | |
| --- | --- | --- | --- | --- | --- |
|  | RR (95% CI) | *P* value |  | RR (95% CI) | *P* value |
| *Age (years)* |  |  |  |  |  |
| ≥ 60 vs. < 60 | 1.128 (0.734 to 1.732) | 0.582 |  | NA | NA |
| *Gender* |  |  |  |  |  |
| Male vs. Female | 1.034 (0.701 to 1.526) | 0.866 |  | NA | NA |
| *Pathological stage* |  |  |  |  |  |
| Ⅲ/Ⅳ vs.Ⅰ/Ⅱ | 2.469 (1.650 to 3.695) | <0.0001 |  | 1.659 (1.009 to 2.726) | 0.046 |
| *T stage* |  |  |  |  |  |
| T3+T4 vs. T1+T2 | 1.839 (1.087 to 3.110) | 0.023 |  | NA | NA |
| *N staging* |  |  |  |  |  |
| Positive vs. Negative | 2.404 (1.639 to 3.528) | <0.0001 |  | 1.791 (1.108 to 2.895) | 0.017 |
| *M stage* |  |  |  |  |  |
| Positive vs. Negative | 1.029 (0.695 to 1.522) | 0.887 |  | NA | NA |
| *PHTF2 expression* |  |  |  |  |  |
| High vs. Low | 1.899 (1.012 to 3.562) | 0.046 |  | 1.392 (0.731 to 2.649) | 0.314 |

NA: not analyzed
